# Using 3PG to assess climate change impacts on management plan optimization of *Eucalyptus* plantations. A case study in Southern Brazil

**DOI:** 10.1038/s41598-021-81907-z

**Published:** 2021-02-01

**Authors:** João HN Palma, Rodrigo Hakamada, Gabriela Gonçalves Moreira, Silvana Nobre, Luiz Carlos Estraviz Rodriguez

**Affiliations:** 1 Forest Research Centre, University of Lisbon, Tapada da Ajuda, 1349-017 Lisboa, Portugal; 2 MVARC-Moinhos de Vento Agroecology Research Centre, 7750-217 Mértola, Espírito Santo Portugal; 3 Department of Forest Science, Federal University of Pernambuco, Campus box: 14098780, Dom Manuel de Medeiros st, Recife, Pernambuco 52171-900 Brazil; 4 Department of Forest Sciences, Escola Superior de Agronomia Luiz Queiroz, Piracicaba, São Paulo 13418-900 Brasil; 5 Atrium Forest Consulting Ltda, Piracicaba, São Paulo Brazil

**Keywords:** Ecological modelling, Forestry, Climate change

## Abstract

*Eucalyptus* plantations around the world have been largely used by the paper industry. Optimizing the management of resources is a common practice in this highly competitive industry and new forest growth models may help to understand the impact of climate change on the decisions of the optimization processes. Current optimized management plans use empirical equations to predict future forest stands growth, and it is currently impractical to replace these empirical equations with physiological models due to data input requirements. In this paper, we present a different approach, by first carrying out a preliminary assessment with the process-based physiological model 3PG to evaluate the growth of *Eucalyptus* stands under climate change predictions. The information supplied by 3PG was then injected as a modifier in the projected yield that feeds the management plan optimizer allowing the interpretation of climate change impacts on the management plan. Modelling results show that although a general increase of rain with climate change is predicted, the distribution throughout the year will not favor the tree growth. On the contrary, rain will increase when it is less needed (summer) and decrease when it is most needed (winter), decreasing forest stand productivity between 3 and 5%, depending on the region and soil. Evaluation of the current optimized plan that kept constant the relation between wood price/cellulose ton shows a variation in different strategic management options and an overall increase of costs in owned areas between 2 and 4%, and a decrease of cumulated net present value, initially at 15% with later stabilization at 6–8%. This is a basic comparison to observe climate change effects; nevertheless, it provides insights into how the entire decision-making process may change due to a reduction in biomass production under future climate scenarios. This work demonstrates the use of physiological models to extract information that could be merged with existing and already implemented empirical models. The methodology may also be considered a preliminary alternative to the complete replacement of empirical models by physiological models. Our approach allows some insight into forest responses to different future climate conditions, something which empirical models are not designed for.

## Introduction

Facing climate change is a current challenge in many economic activities in the world. Amongst such activities are those directly related to forest growth which directly depends on climatic conditions. Nearly 4 billion hectares covered by forests in the world and about 280 million belong to the so-called planted forests^1^. In Brazil, planted forests occupy 10 million hectares and represent 1.2% of the national territory and 2% of the forest area^2^. Planted forests have a high socioeconomic relevance because they represent about 90% of the wood extracted in Brazil^2^, with about 250 million cubic meters extracted annually. A good part of this volume plays an environmental role, by reducing the extraction of wood that would previously have been extracted from native forests^3^. In Sao Paulo State, where the study was carried out, *Eucalyptus* plantation covers 918 thousand hectares, representing by 12% of the total area within the country^2^. *Eucalyptus* is one of the most commonly used species to provide wood biomass for the paper industry worldwide, and the response of tree growth under climate change is needed to understand the impacts that may occur in the needs of paper mills. Furthermore, industrial plantations may play an important role regarding the last decades of land use change. For example, in the Paraná river basin in Brazil has been suffering tremendous land use change in the last decades. Since 1965, forest cover decreased from 24 to 5% in 1990, being replaced by annual crops since the 1970s, causing an increase of water discharge of the Paraná river^4^. Therefore, forest plantations may buffer the discharge into Paraná river. However, plantations need to be sustainable and accounting for climate change in the management plan is crucial for sustainability of such plantations in long term.

While several artworks have studied management regimes and productivity responses of *Eucalyptus* in Brazil^5–8^, the assessment of climate change impacts on *Eucalyptus* plantation productivity is needed to support decisions concerning strategies of future plantations.

The assessment of climate change impacts on forest productivity can be explored with the help of process-based models because these models use climate inputs as drivers for the estimation of available resources needed for forest growth^9^. The 3PG model has been widely tested to estimate productivity for the most planted genus worldwide as *Pinus*^10^, *Eucalyptus*^11,12^, and *Cunninghamia*^13^.

Because of the worldwide use of *Eucalyptus* plantations, assessments of productivity under climate change scenarios have been done in different parts of the globe^14^. A focus on the Brazilian context predicts, in the medium-long term, productivity losses of up to 40% in the period 2071–2100^15^ and a reduction in forest area by up to 82% in certain regions^16^. However, none of these studies are linked to application within a real case study of an industry using models and tools to predict forest growth, where the projections feed the elaboration of a strategic optimized management plan.

Forest management planning and its optimization are as common and wide as industrial plantations are. Most of the forest management optimization plans around the world have been using empirical models to supply the biophysical data (forest yield) needed to feed the mathematical models that optimize the management goals (e.g. maximization of profit) within associated constraints (e.g. even flow of wood to the mill). This work proposes a method of how such optimization procedures can be improved by supplying information from process based models that are capable of estimating the impact of climate change on forest yields, and therefore impacting on the strategic plans and helping in the decision-making process. For demonstration purposes, such a procedure is presented here with a case study, but the method can be widely transferrable to any forest plantation in the world which is under an optimized management plan.

The optimized management plan being used in this case study covers about one hundred thousand hectares of land in Brazil and is managed by the multinational “International Paper Brasil”, thereafter called “Company”. The plan minimizes the cellulose cost per unit, requiring an even flow of biomass entering the mill, and provides information for the harvesting schedule in the field as well as a cost–benefit analysis of the entire system, with different intermediate decision options and indicators.

As part of an EU International Research Staff Exchange Scheme (FOREADAPT—Knowledge Exchange between Europe and America on Forest Growth models and Optimization for Adaptive Forestry), this work tests the potential of using the outputs of the 3PG physiological model^9^, which projects forest growth under climate change, in the management optimization algorithms, and observes the subsequent changes in the strategy of the optimized management plan of the Company.

## Materials and methods

### Climate soil and management data

To assess climate change effects on forest growth, future climate scenarios were obtained through the Instituto Nacional de Pesquisa Espacial (INPE). The Company assessment area is comprised of five geographically distinct areas. For each area, the centroid coordinate was given to INPE, resulting in five different grid cells of the downscaled HadCM3 dataset ^17,18^, with the scenario A1B of the International Panel on Climate Change (IPCC). Scenario A1B is similar to the representative concentration pathway 6 (RCP6), between RCP4.5 and RCP8.5^19,20^. For each coordinate, two datasets were extracted; a control dataset (CNTRL) with the current climate and a dataset with the median level of climate change of the scenario A1B (MIDI). An attempt to identify water stress days was calculated, but little is known about this indicator because it is related to hydraulic tension in the soil and varies depending on soil characteristics and the thematic study (e.g. fire occurrence). Nevertheless, we considered a “water stress day” if the sum of precipitation of the previous 15 days was lower than 10 mm; however, this indicator was not used for any modelling purposes but rather for supplying extra information for interpreting the differences between current and future climates. Monthly data was calculated and averages for a 30-year timeframe were gathered and formatted for input to 3PG.

For each of the five regions, there were two main soil types: (1) Sandy soil, 3 m deep and (2) Clay soils, 10 m deep, and the percentage of each soil type in each region varied according to Table 1.

**Table 1:** Centroid coordinates and soil type coverage from each of the regions representative of the study.

Region	Approximate location		% Soil type in region	
	Latitude	Longitude	Sandy average depth: 3 m	Clay average depth:10 m
A—Mogi Guaçu	− 22.2070	− 46.9700	30	70
B—Brotas	− 22.1892	− 48.0879	30	70
C—São Simão	− 21.5827	− 47.5715	65	35
D—Luis António	− 21.1120	− 47.0409	65	35
E—Poço de Caldas	− 21.8761	− 46.5690	50	50

### 3PG modeling setup

The 3PG model was previously calibrated specifically for the studied regions by Baesso et al.^15^ and therefore was ready to reproduce the *Eucalyptus* growth in the study areas. The projections of forest growth for each of the five regions were set for the sandy and clay soils, a tree density plantation of 1212 trees ha^−1^ and a final cut at 7 years. For each of the simulations the current climate (CNTRL) and future climate (MIDI) was set, providing a total of 20 simulations for analysis.

Each of the regions was compared independently, and yield differences estimated with the model for each region were calculated proportionally to the soil type coverage. Finally, for each region the differences between current and future climates were compared.

### Linkage with the optimization procedure

The optimization procedure is currently done with WoodStock^21^, a software that organizes data to produce a mathematical equation to be solved through linear programming by a solver (e.g. CPLEX^22^) and interprets the optimized results.

WoodStock^21^ is currently linked to the forest inventory database where data is retrieved to be projected with empirical growth models. During the process, yield tables are generated with the growth models that feed the forest productivity of the algorithm.

The WoodStock^21^ procedure involves a specific interface language to produce the mathematical equation to solve and within this already-developed algorithm by the Company, a section is dedicated to genetic improvements. This section is composed of a few lines where a yearly correction factor of 1.x (“x” being the increase), is applied to the yield tables, to adjust the yearly predicted genetic improvement program.

With the same rationale, a section with a correction factor for the impact of climate change was developed. The correction factor was the difference in yields estimated earlier with the 3PG model. There were five sections, one dedicated to each region.

For clarity of results regarding the biophysical impact on the current optimization plan, the authors made no changes in the existing financial/economic model set up being carried out in the Company.

WoodStock^21^ generated the inputs to the optimizer (CPLEX^22^). The solving took about 75 min (Intel i7 Quad Core Processor, 2.6Ghz with turbo-boost up to 3.3 Ghz). Optimizer results were processed again by WoodStock^21^ and outputs generated and integrated into MSExcel for graphical output.

### Insights to undergoing management plan

Currently the forest management plan has three essential pillars. The first is the division in terms of forest ownership: (1) owned land, (2) in partnership and (3) Landowner Assistance Program (LOAP). The second is the permanent investment in genetic resources improvement. The third is a dynamic compensation program of land acquisition and wood sale either from owned or not owned areas. The entire management goal is to keep constant the relation Cost of Wood/Cellulose ton, which, in optimization terms, is a constraint to the mathematical problem.

## Results and discussion

Studies on assessment of optimized strategic management planning under climate change at landscape scale are scarce. While^23,24^ studied the impact of future climate change in optimized forest management in the Boreal and Mediterranean context, some^25^ used a similar approach as the present work by linking empirical tree-level models with transfer variables fed from process based models to include climate change in large-scale forest scenario analysis.

These studies envisage the assessment of climate change on optimized forest productivity. However, they miss the objectives proposed in the present work to bridge interaction with an industrial real/practical case study where more variables than sustainable harvested volume and net present value have to be considered for strategic decisions to be taken, in reaction to possible changes in forest productivity due to climate change. The main difference of the present work is not only the estimation of the impact of climate change on sustainable forest productivity but rather the observations on how such a different sustainability scenario (forced by climate change) influences the downstream decision-making processes.

The drawback of the assessment in a real case study is the difficulty to compare the results to other case studies, because the detail at this level is so specific for the case study envisaged that there will be virtually no comparison possible regarding the interpretation of the behavior of the decision variables. However, the most important aspect of this assessment is the methodology behind the link between a process-based model and an existing optimization procedure that currently does not take climate change into consideration. Nevertheless, the results here presented are valid to understand the dynamics of decisions that are related to changes in forest productivity, either for climate change or another driver.

### Climate

**Figure 1:**
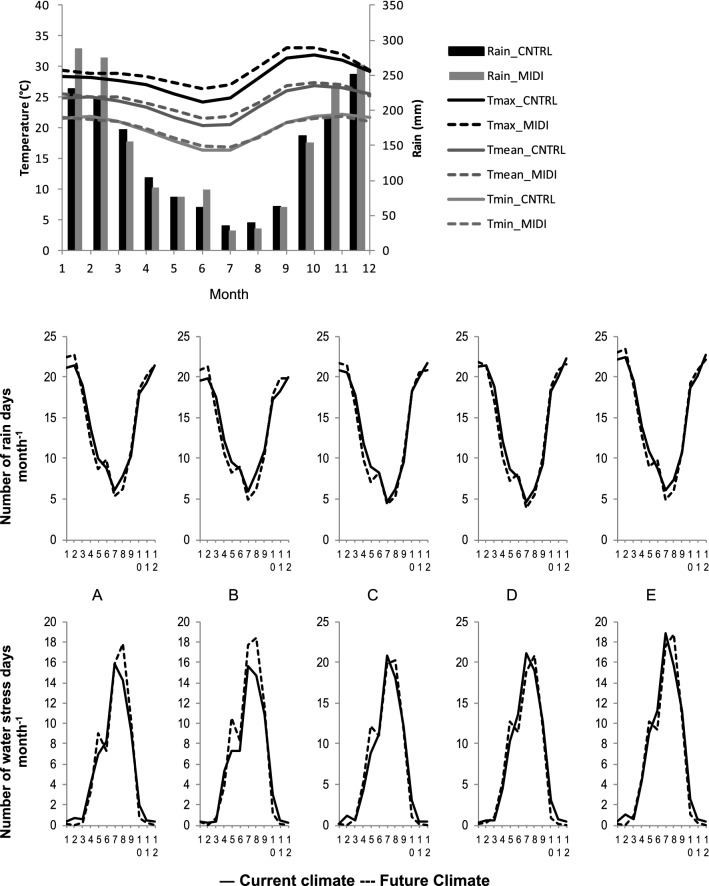
Brotas (region B), comparison of 30 years data (2011–2040) of current (CNTRL) and future (MIDI) minimum, maximum and mean temperature (T_min_, T_max_, T_mean_) and Rain. Number of rain days and water stress days per month regions (**A**) Mogi Guaçu, (**B**) Brotas, (**C**) São Simão, (**D**) Luís Antonio and (**E**) Poços de Caldas. Created using Excel 2019 (https://www.microsoft.com/pt-br/).

All five regions have similar differences between current and future climates, and only the region of Brotas is shown in full detail (Fig. 1). The comparison shows that temperature will rise, especially during the winter. Regarding rain, predictions suggest an overall increase, however the increase is concentrated in the summer. These differences combined are of great importance for tree growth. On one hand, more rain will occur when it is less needed (summer) because water is not a limiting factor in this season. On the other hand, less rain will occur when there is already water deficit (winter). Furthermore, the increase of temperature is directly related with an increase of vapour pressure deficit^26,27^, one of the main drivers for evapotranspiration and tree growth. Therefore, although the rise in temperature could be important to increase tree growth, this growth will stand as potential and not actual growth because water will be limiting growth.

### Tree growth projections

**Figure 2:**
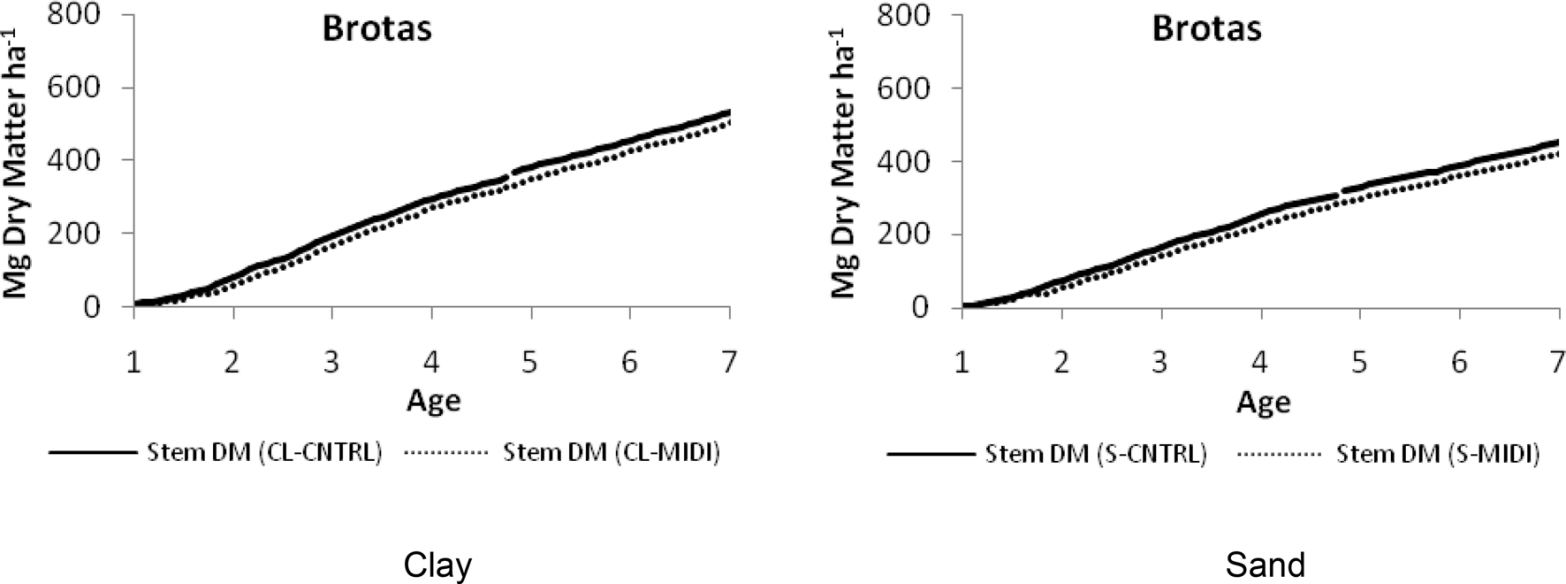
Comparison of stem dry matter (DM) for 7 years rotation under current (CNTRL) and future (MIDI) climate for clay (CL) and sandy (S) soils in Brotas.

Because the differences between current and future climate are similar between the five regions, a corresponding tree growth difference was expected and found. All the remaining regions had similar growth differences Again, the graphical results displayed are simplified by showing only the region of Brotas—Fig. 2. Each of the five regions had clay and sandy soils in a certain proportion and the representative differences in yield were calculated with a weighted average, with the proportion of the soil types as the weight in the calculation. The range of differences between all the regions and soil types varied between 2 and 6% and, when averaged, the range became 3–5% in yield reduction (Table 2). These results are in line with findings of some studies^27^ and with works that suggest that increased drought is likely to lead to reduced plant growth and primary productivity^28^.

**Table 2:** Biomass difference in the final year of rotation under climate change for each region and soil type.

Region	Soil type	Biomass difference in year 7	% soil type in region	Weighted average of reduction (%)
A—Mogi Guaçu	CL	0.96	70	4.0
	S	0.97	30	
B—Brotas	CL	0.95	70	4.9
	S	0.94	30	
C—São Simão	CL	0.98	35	2.9
	S	0.97	65	
D—Luis Antonio	CL	0.97	35	3.4
	S	0.96	65	
E—Poço de Caldas	CL	0.97	50	3.0
	S	0.97	50	

*S* sandy, *CL* clay.

### Impact on the optimized management plan

**Figure 3:**
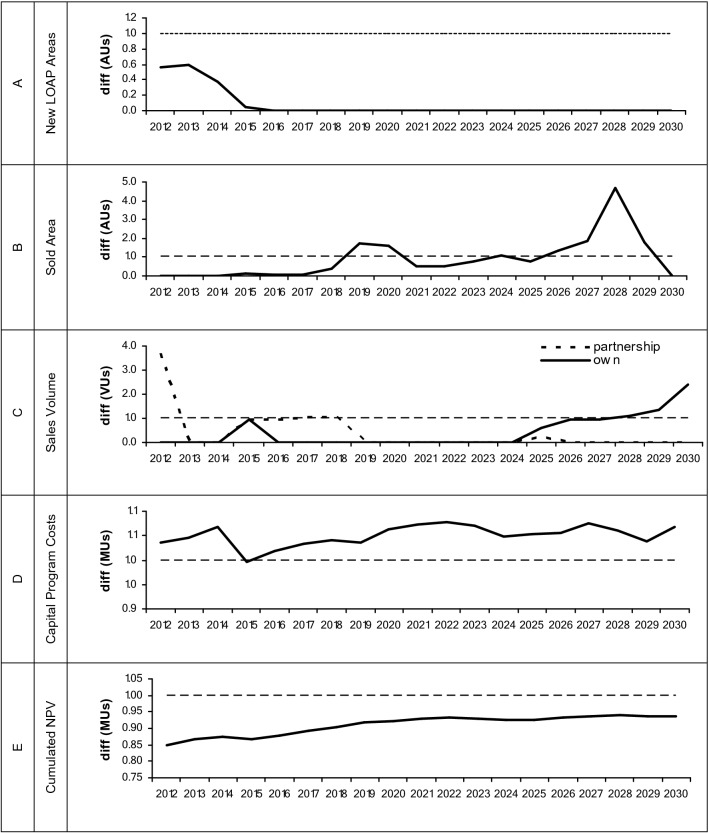
Main differences in the optimization management plans between current climate (dash line reference) and future climate on (**A**) Acquisition of new LOAP Areas, (**B**) Sold Area, (**C**) Sales Volume, (**D**) Capital Program Costs, (**E**) Cumulated Net Present Value. *AUs* area units, *VUs* volume units, *MU’s* monetary units.

Numerous accounting and decision variables are calculated during the optimization procedure. We synthesize some of the variables in Fig. 3 where major differences were found. We focus on (1) New LOAP areas, (2) Sold Area, (3) Sales Volume, (4) Capital Program Costs and (5) Cumulated Net Present Value.

The impact of climate change and the reduction of productivity of in the range 3–5% (Fig. 2) reflects differences in different strategic options to keep constant the ratio between cost of wood per ton of cellulose. The first, not ordered by strategic importance, is that the acquisition of new LOAP areas should immediately decrease (Fig. 3A). This decision option is strongly related to the reduction of sold area in the first third of the planning horizon (Fig. 3B), highlighting the importance of keeping owned areas.

A possible interpretation of this optimized strategy is to avoid loss and, when possible, increase access to the genetic material when planting new material. In this way, future forest yield is better controlled.

As an additional strategy to both decreasing new LOAP areas and avoiding selling owned land, there should be an increase in wood sold from partnership forests right in the beginning and during the first third of the planning horizon. The reduction of the selling area is only postponed until the last third of the planning horizon (Fig. 3B), which is related to a higher sales volume from owned forest in that period (Fig. 3C).

Arguably, the most important indicators are financial. Although keeping the ratio Cost of wood/Cellulose ton a constant throughout the planning horizon, the impact of climate change in wood productivity has associated costs. The capital program costs (costs of planting, coppice, and maintenance) have an overall increase of about 10%—Fig. 3D. An interpretation of this increase could be the increased owned plantations to manage and the investment in the genetic improvement program that seems to be the key for the adaptive management needed to avoid larger losses.

The whole net present value of the system seems to suffer in two ways. In the first stages there is a higher drop in return of 15%. Then, it starts to gently recover and keep steady at about 6–8% of the current climate scenario. This behavior could be explained with two distinct phases. One, at the beginning, that avoids the returns by selling owned land, and a second stage where the recovery starts when new plantations, with new genetic material, starts to become effective.

Changing the procedures of using empirical forest growth projections in the current management planning may not be an immediate option due to the complexity and data requirements of process-based models. This work envisaged an intermediate assessment where the process-based 3PG model supplies information on differences in forest growth under current and future climates which, à posteriori, can be merged as modifiers in the empirical growth curves used by the optimizer.

**Figure 4:**
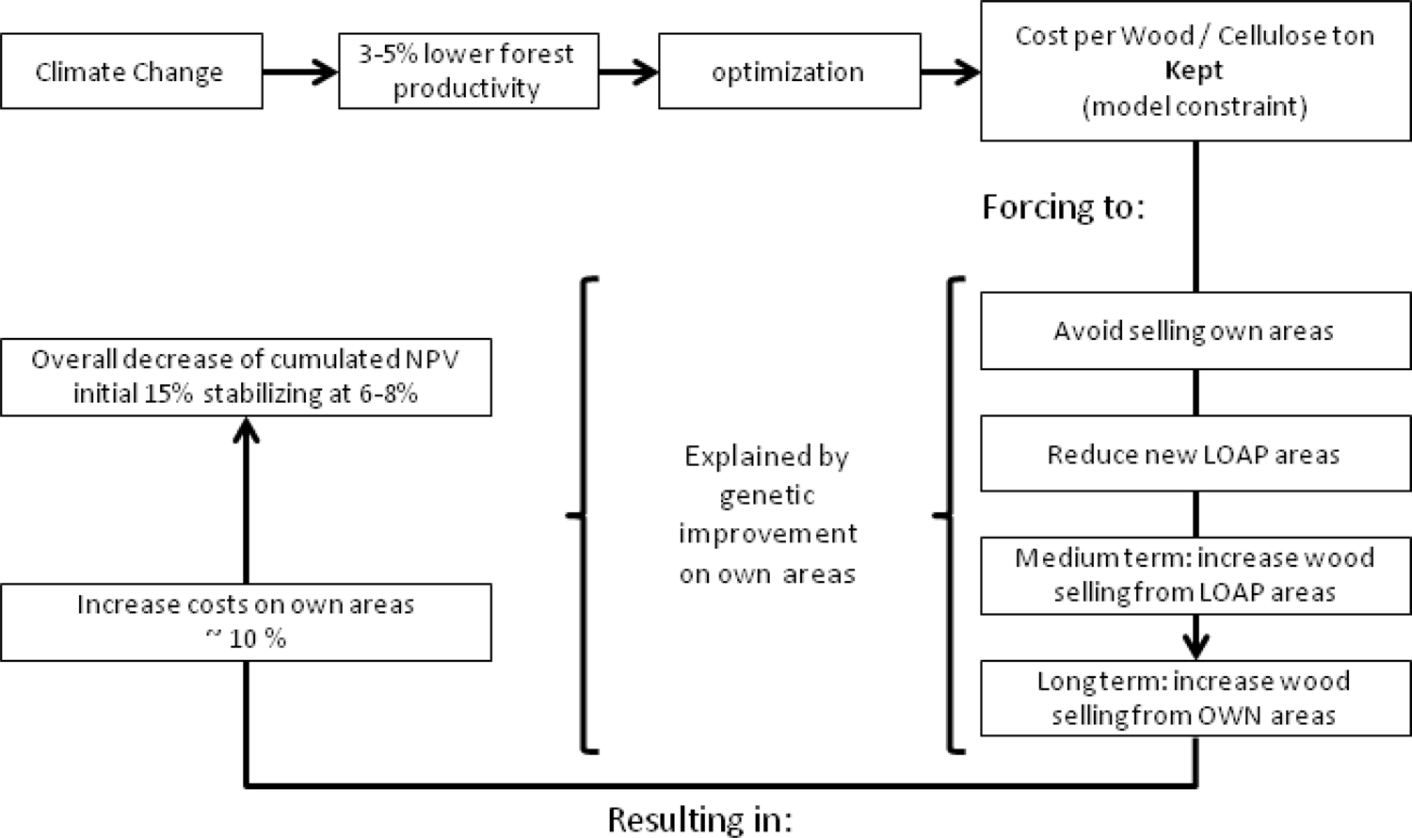
Overview of the effect of climate change on the forest management plan optimization dynamics.

The effects of climate change on the optimized management plan in this case study is presented in Fig. 4, where the dynamics of the system affected by climate change is represented.

Although the ultimate objective was to assess the impact of climate change in the future unitary cost of cellulose entering the pulp mill of this case study, the methodology provided here can be reproduced anywhere (with slightly adaptations depending on software usage). This methodology could be particular useful if the use of physiological models is not a viable option due to procedural integration of complexity, or data requirements, needed to supply the new model.
